# The Association of Altered Gut Microbiota and Intestinal Mucosal Barrier Integrity in Mice With Heroin Dependence

**DOI:** 10.3389/fnut.2021.765414

**Published:** 2021-11-04

**Authors:** Jiqing Yang, Pu Xiong, Ling Bai, Zunyue Zhang, Yong Zhou, Cheng Chen, Zhenrong Xie, Yu Xu, Minghui Chen, Huawei Wang, Mei Zhu, Juehua Yu, Kunhua Wang

**Affiliations:** ^1^Faculty of Life Science and Technology, Kunming University of Science and Technology, Kunming, China; ^2^Department of Clinical Laboratory, The First Affiliated Hospital of Kunming Medical University, Kunming, China; ^3^Medical School, Kunming University of Science and Technology, Kunming, China; ^4^National Health Commission (NHC) Key Laboratory of Drug Addiction Medicine, First Affiliated Hospital of Kunming Medical University, Kunming, China; ^5^Centre for Experimental Studies and Research, First Affiliated Hospital of Kunming Medical University, Kunming, China; ^6^Department of Gastrointestinal Surgery, The First Affiliated Hospital of Kunming Medical University, Kunming, China; ^7^Department of Administrative Affairs, Yunnan University, Kunming, China

**Keywords:** heroin dependence, gut microbiota, intestinal mucosal barrier integrity, SCFAs, mice

## Abstract

The gut microbiota is believed to play a significant role in psychological and gastrointestinal symptoms in heroin addicts. However, the underlying mechanism remains largely unknown. We show here that heroin addicts had a decrease in body mass index (BMI) and abnormal serum D-lactic acid (DLA), endotoxin (ET) and diamine oxidase (DAO) levels during their withdrawal stage, suggesting a potential intestinal injury. The gut microbial profiles in the mouse model with heroin dependence showed slightly decreased alpha diversity, as well as higher levels of *Bifidobacterium* and *Sutterella* and a decrease in *Akkermansia* at genus level compared to the control group. Fecal microbiota transplantation (FMT) further confirmed that the microbiota altered by heroin dependence was sufficient to impair body weight and intestinal mucosal barrier integrity in recipient mice. Moreover, short-chain fatty acids (SCFAs) profiling revealed that microbiota-derived propionic acid significantly decreased in heroin dependent mice compared to controls. Overall, our study shows that heroin dependence significantly altered gut microbiota and impaired intestinal mucosal barrier integrity in mice, highlighting the role of the gut microbiota in substance use disorders and the pathophysiology of withdrawal symptoms.

## Introduction

As a chronic and relapsing brain disease (1, 2), heroin addiction is usually characterized by sensitization, dependence, and compulsive drug use (3, 4). Beyond neuropsychiatric disorders, our previous studies showed that heroin abuse is often associated with decreased appetite, constipation, nutritional and gastrointestinal symptoms that can lead to low abstinence rate and malnutrition or nutritional risks during the withdrawal period (5–7). More seriously, drug-induced intestinal barrier lesions and severe enterogenic infections or related death were observed in Rhesus monkeys (5) and methamphetamine treated mice (8). Notably, lower zona occludens-1 (ZO-1) immune positivity was observed in heroin abusers compared to healthy controls (9), indicating that heroin affects the molecular integrity of tight junction (TJ) proteins, which correlates with gastrointestinal symptoms.

Although the mechanism of the gut microbiota in disease is not fully understood, the gut microbiota is a critical component of the brain-gut axis (10) potentially impacting behavior and mood (11), key modulators of host immunity (12), metabolic (13) and other diseases (14, 15). The role of gut dysbiosis is considered an essential factor in the development of drug addiction (10, 16). Furthermore, gastrointestinal disorders may be accompanied by gut microbiota dysbiosis (17). However, the association between gut microbiota and intestinal mucosa barrier function in the context of substance use disorder remains largely unknown. Further investigation is urgently needed to uncover the roles of intestinal microbiota in gastrointestinal symptoms in heroin addicts.

In this study, we investigated the serum indicators of intestinal mucosal barrier damage in heroin addicts during withdrawal stage and generated a mouse model mimicking heroin dependence. High-throughput sequencing of 16s rRNA gene and bioinformatic analysis was performed to determine the variations in gut microbiota in heroin dependent mice compared with saline-treated mice. To confirm the altered microbiota contributing to the intestinal mucosal barrier integrity, donor stool from mice with heroin dependence were transplanted into recipient mice by fecal microbiota transplantation (FMT). To assess intestinal mucosal barrier integrity, the serum level of DAO, DLA, and ET, as well as protein expression levels of Claudin-1 and ZO-1 were evaluated, respectively. The concentrations of metabolic products SCFAs in mouse fecal samples were analyzed using gas chromatography–mass spectrometry (GC-MS).

We show that heroin dependence causes substantial changes in the composition of the gut microbiota contributes to pathological changes of intestinal mucosal barrier function. Furthermore, it offers a deep insight into the function of the gut microbiota in heroin dependence induced digestive diseases and a new paradigm for addiction treatment.

## Materials and Methods

### Study Populations and Ethics Statement

Thirty-eight male heroin addicts (age ranging from 18 to 58) from the hospital of the Longchuan Drug Rehabilitation Center in Dehong, China were recruited as the heroin group; 38 age-matched males with no heroin-use history were recruited as healthy controls (HCs). The inclusion criteria were: (1) male sex and aged between 18 and 58, (2) diagnosed with heroin dependence, and (3) in withdrawal stage. Participants were excluded if (1) severe neurological disorders, (2) severe chronic illnesses, infections, (3) antibiotic treatments (<7 days prior to study start), (4) refuse or inability to give informed consent. All participants provided written informed consent prior to study participation. The participants' age, gender, body weight, and height were collected. 2 ml of blood was drawn from each participant to measure intestinal mucosal barrier damage indicator.

### Animals

Male C57BL/6 SPF mice (6–8-week-old, weighing 20–25 g) were obtained from the Kunming Medical University Center for Laboratory Animals, housed individually under controlled temperature (22 ± 2°C) and humidity (50 ± 5%), and maintained on a 12/12 h light/dark cycle (lights on at 7 a.m.). The mice had free access to tap water and a standard mouse chow diet. Animals were deeply anesthetized with pentobarbital sodium (40 mg/kg, i.p.) then sacrificed, colonic tissues were perfused with 0.1 M PBS (pH 7.4, 37°C) followed by 4% (w/v) paraformaldehyde in 0.1 M PBS, prepared at 20 μm thickness by using a cryostat microtome (Leica CM 3500, Wetzlar, Germany). All experiments were conducted in accordance with the National Institutes of Health Guide for the Care and Use of Laboratory Animals.

### Drugs

The Narcotics Department of Yunnan Provincial Public Security Administration generously supplied the diacetylmorphine HCl (heroin), and the heroin was freshly dissolved in 0.9% sterile saline for intraperitoneal (10 mg/kg).

### Experimental Design

**Figure 1:**
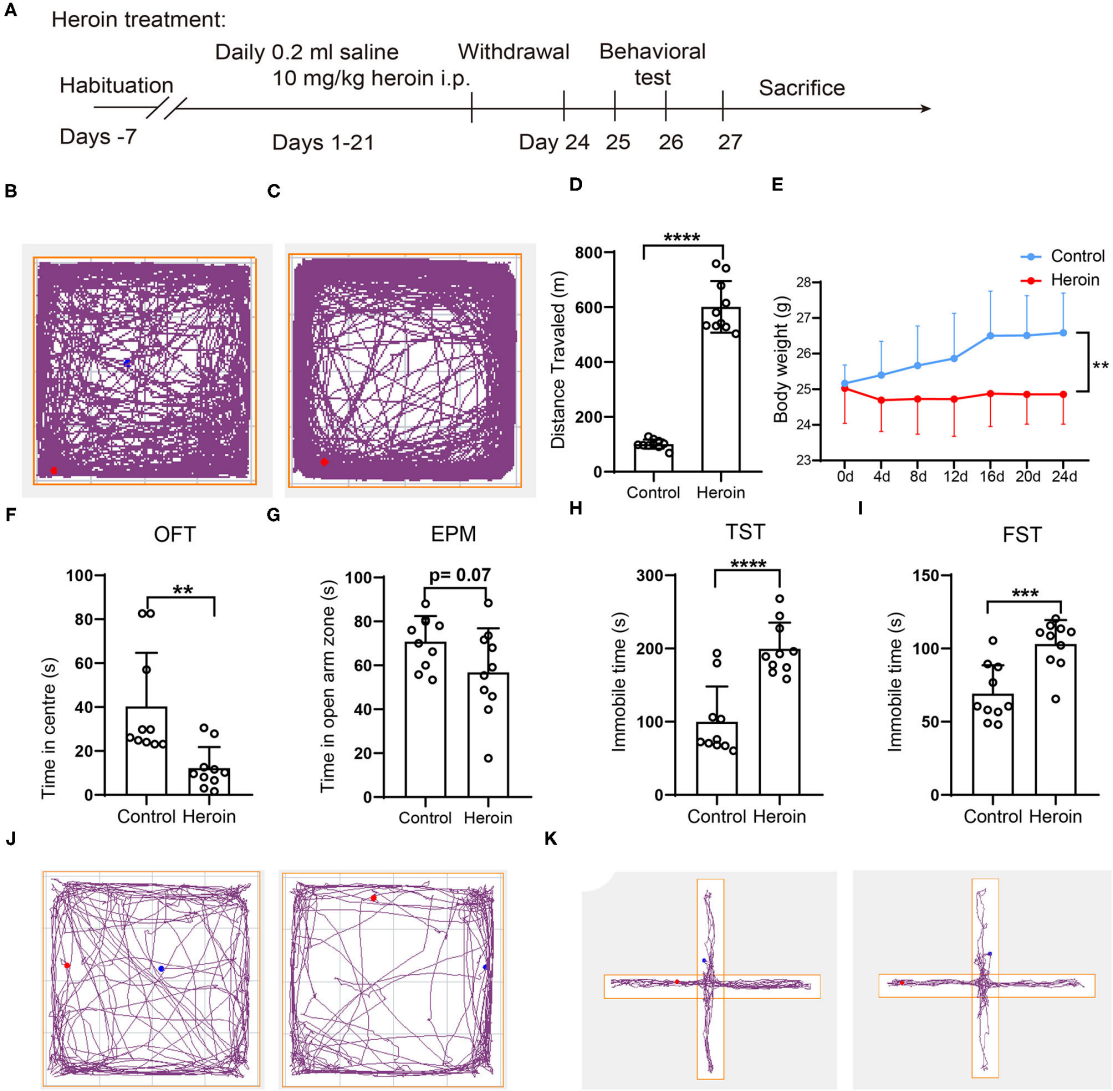
Heroin-induced mouse behavior sensitization, neurobehavioral deficits, and body weight loss in C57/BL mice. **(A)** experimental design: C57/BL mice were administered an intraperitoneal (i.p.) injection of 10 mg/kg heroin for 21 days. After a 48-h withdrawal period, fresh fecal samples were collected for 16s rRNA sequencing, and cecal tissues were collected after sacrifice. **(B–D)** A 10 mg/kg heroin administration induced behavior sensitization. Track plots showing the position of the animal for 60-min tests where treatment with control **(B)** and heroin **(C)** (*n* =10); **(D)** Bar graphs show total distance traveled of the open field during the test. **(E)** Heroin- administrated mice showed reduced body weight compared to controls. **(F–K)** heroin-administrated mice exhibit anxiety and depression-like behavioral deficits: **(F)** time spent in the central area of the open field during the test; **(G)** time spent in open arms of the elevated plus maze; **(H)** Immobility time during the tail suspension test; **(I)** Immobility time in the forced swim test; **(J)**Track plots showing the position of the animal for 5-min tests in OFT where treatment with saline (left) and heroin (right); **(K)** Track plots showing the position of the animal for 5-min tests in EMP where treatment with saline (left) and heroin (right) (*n* = 10). The student's *t*-test was used to determine whether differences existed between the two groups, **p < 0.01; ***p < 0.001; ****p < 0.0001. All data are expressed as the mean ± SD.

**Figure 2:**
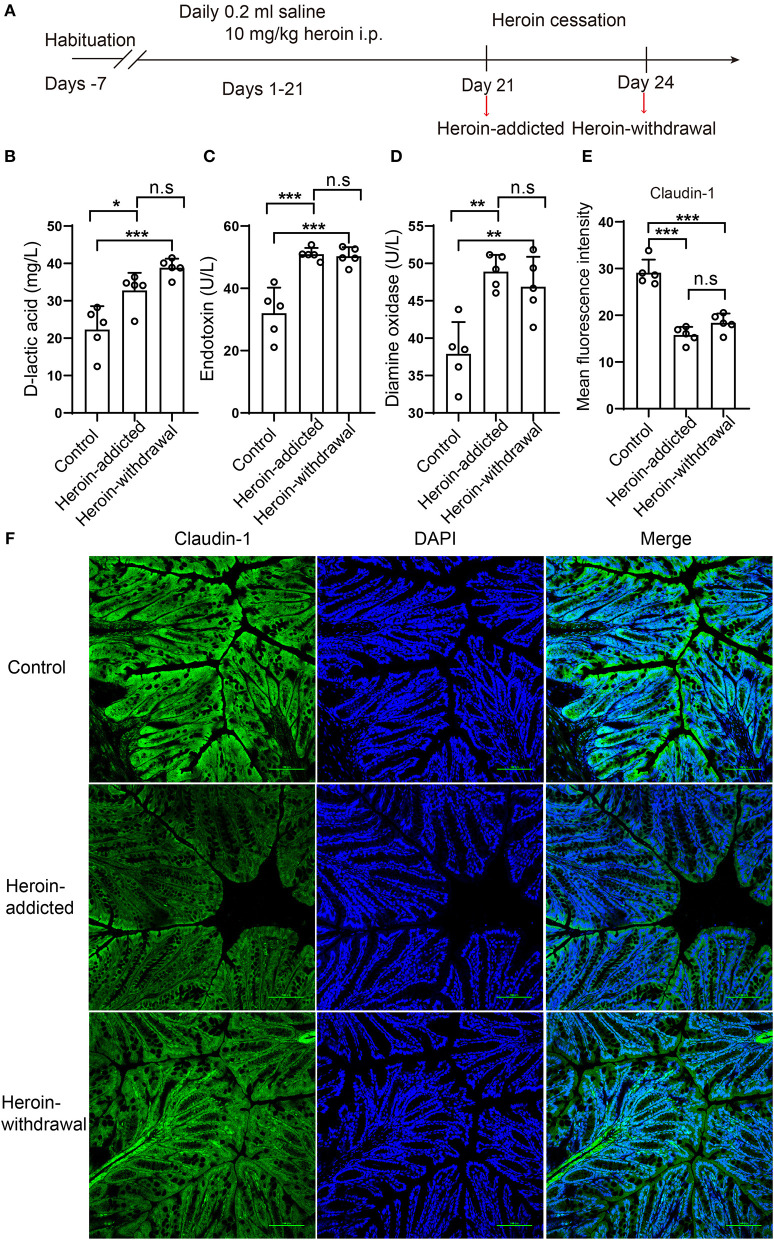
Disrupted the intestinal mucosal integrity in mice with heroin dependence. **(A)** experimental design. The serum and colon tissues were collected on day 21 as heroin-addicted group, and after a 48-h withdrawal period (on day 24) as heroin-withdrawal group. **(B–D)** The serum levels of D-lactic acid (DLA), endotoxin (ET) and diamine oxidase (DAO) in heroin-addicted, heroin- withdrawal and control mice. **(E)** Immunofluorescence showed decreased expression levels of Claudin-1 in heroin-addicted and heroin-withdrawal groups. **(F)** Mean fluorescence intensity of Claudin-1 expression. All data are expressed as the mean ± SD *p < 0.05; **p < 0.01; ***p < 0.001 (*n* = 5 in each group).

In order to generation the mouse model with heroin dependence, mice were randomly separated into two groups after 1 week of adaptation: (1) control group (0.2 ml saline i.p. *n* = 10); (2) heroin group (10 mg/kg heroin i.p. *n* = 10). The experimental design is showing in Figure 1A. Mice were treated with 10 mg/kg of heroin or saline for 21 continuous days. After 48 h withdrawal (day 24), fecal were collected and behavioral tests were performed. Mouse weight was periodically monitored every fourth day during the heroin treated, thus we collected the body weight on day 0–4–8–2–16–20–24, respectively. The intestinal mucosal integrity was investigated and heroin dependent mice were sacrificed at days 21 (Heroin-addicted) group and at days 24 (Heroin-withdrawal) group (Figure 2A).

### Behavioral Locomotor Sensitization

Heroin-induced behavioral sensitization, which indicated addiction behavior, was measured using an open field test (OFT) and the ANY-maze software (Stoelting Co.) (18). Mice were placed individually in the corner of an open-field box (45 × 45 × 30 cm) and allowed to explore for 1 h in the OFT apparatus after heroin/saline treatment. The tracked plot and total distance traveled were collected and analyzed using a video tracking system (ANY-maze, Stoelting Co.).

### Fecal Microbiota Transplantation (FMT)

We performed FMT as described previously (19, 20): fecal pellets (collected on day 24) from different mice in the corresponding group were resuspended together in PBS (1 fecal pellet/ml) for about 15 min, shaken and then centrifuged at 1,000 rpm, 4°C for 5 min. The suspension was centrifuged at 8,000 rpm, 4°C for 5 min to get total bacteria, then filtered twice in PBS. The final bacterial suspension was mixed with an equal volume of 40% sterile glycerol to a final concentration of 20%, then stored at −80°C until transplantation. The recipient mice (SPF male C57/BL/6) were gavaged with 200 μl of the fecal slurry or PBS once a day for seven consecutive days. Body weight changes of recipient mice in pre-FMT and post-FMT were monitored, using pre-FMT body weight as baseline for FMT. A total of 30 mice were used in FMT experiment: donor mice (administrated with heroin for 21 days and collected fecal to prepare bacterial suspension *n* =1 0), recipient mice: (gavaged with 200 μl of the fecal slurry *n* = 10, gavaged with 200 μl of PBS *n* = 10).

### Open-Field Test (OFT)

The OFT assessed the effects of chronic psychological stress on anxious behavior. The mice were subjected to OFT for 5 min. The time spent in the center (inner 25% of the surface area) were recorded by ANY-maze software (Stoelting Co.) The open field arena was cleaned with 70% ethanol between each trial.

### Elevated Plus-Maze Test (EPM)

The elevated plus-maze test was performed as previously described (21). The apparatus includes two open arms, two enclosed arms, and a central platform. The entire device was elevated 45 cm above the floor. A mouse was placed on the central platform and allowed to roam freely for 5 min. The time spent on open and closed arms were recorded.

### Forced Swimming Test (FST)

A day before the test, a mouse was placed individually into a glass cylinder (20 cm height, 17 cm diameter) filled with water to a depth of 10 cm at 25°C for 5 min. During experimentation, subjects were placed individually in a filled glass cylinder for a period of 6 min and the total duration of immobility was measured for 5 min by ANY-maze software as indicative of depressive-like behavior. Motionless floating was considered immobile behavior (21).

### Tail Suspension Test (TST)

The tail suspension test was conducted as described previously (21). Mouse was suspended 60 cm above the surface by adhesive the tip of the tail. Duration of immobility in a 6-min period was recorded by a video tracking system (ANY-maze, Stoelting Co.), which was indicative of depressive-like behavior.

### Fecal Sample Collection and Microbial DNA Extraction

Fecal samples were collected from the control and heroin dependent mice, frozen immediately and stored at −80°C. Microbial DNA was extracted from fecal samples fusing the MagPure Stool DNA KFKit B (Magen, China). The quantity of genomic DNA was verified using the Qubit dsDNA BR Assay kit (Invitrogen, USA).

### 16S rRNA Gene Sequencing and Bioinformatic Analysis

16S rRNA gene sequencing procedure was performed by BGI (Shenzhen, China). Briefly, the V4 regions of the 16S rRNA gene in the DNA extracted from fecal samples were amplified using the following degenerate PCR primers: 515F (5′-GTGCCAGCMGCCGCGGTAA−3′), 806R (5′- GGACTACHVGGGTWTCTAAT-3′) and sequenced on the Illumina HiSeq2500 platform following the standard pipelines of Illumina and generating 2 × 250 bp paired-end reads. Taxonomic annotation was based on a customized version of SILVA reference database. Alpha and beta diversities were analyzed using QIIME. The relative abundance of bacteria was expressed as the percentage. Partial least-squares discrimination analysis (PLS-DA) was performed using the R package mix Omics, whereas principal coordinate analysis (PCoA) was performed using QIIME.

### The Serum Levels of D-Lactic Acid (DLA), and Endotoxin (ET) and Diamine Oxidase (DAO)

The serum levels of DLA, ET, and DAO reflect intestinal mechanical barrier dysfunction, usually regarded as a very important evaluation of intestinal mucosal barrier damage (5). The indicators were determined by a combined test kit developed by the Institute of Biophysics, Chinese Academy of Sciences (Beijing Zhongsheng Jinyu Diagnostic Technology Co., Ltd.).

### Immunofluorescence Staining

Expressions of Claudin-1 and ZO-1 were evaluated with immunofluorescence staining. Briefly, mouse colon tissue was fixed in 4% paraformaldehyde for 24 h, dehydrated using 30% sucrose, embedded in frozen section embedding agent and sliced (10-microns thick), permeabilized using 0.3% triton X-100 for 10 min, and blocked with 10% BSA for 60 min. The following primary antibodies was used: Claudin-1 (1:100, Abcam #ab15098), ZO-1 (1:200, Abcam #ab96587). The tissue sections were then incubated with the appropriate Anti-rabbit IgG (H+L) or F(ab')2 Fragment (Alexa Fluor® 488 Conjugate) (1:1000, CST#4412). Nuclei were stained with DAPI. The immunofluorescence quantification was analyzed by image J. Each experiment was repeated three times.

### Detection of SCFAs in Fecal Samples

SCFAs contents were detected using MetWare (http://www.metware.cn/) based on the Agilent 7890B-7000D GC-MS/MS platform. As previously described (22), 20 mg of fecal samples were accurately weighed and placed in a 2-ml EP tube. A total of 1 ml of phosphoric acid (0.5% v/v) solution and a small steel ball were added to the EP tube. The mixture was grinded for 10 s, three times, then vortexed for 10 min and ultrasonicated for 5 min. A total of 0.1 mL supernatant was added to a 1.5-mL centrifugal tube after the mixture was centrifuged at 12,000 rpm for 10 min at 4°C and 0.5 mL MTBE (containing internal standard) solution was added to the centrifuge tube. The mixture was vortexed for 3 min and ultrasonicated for 5 min. Subsequently, the mixture was centrifuged at 12,000 rpm for 10 min at 4°C, and the supernatant was collected and used for GC-MS/MS analysis.

### Statistical Analysis

The data were analyzed using the GraphPad Prism v.8 Software, data reported as means ± SD. Student's *t*-test and a Wilcoxon-test were performed to determine whether differences existed between the two groups. One-way ANOVA was used to compare between three or more groups. A *p*-value of < 0.05 was considered statistically significant (^*^p < 0.05; ^**^p < 0.01; ^***^p < 0.001; ^****^p < 0.0001). Notable non-significant differences were indicated in the figures by “n.s.,” non-near significant (0.05 <*p* < 0.1) were indicated in the figures.

## Results

### Subject Characteristics

A total of 76 participants (38 healthy controls and 38 heroin addicts) were included in the survey sample, and population demographics revealed that the distribution of the sample population by sex and age was not statistically different between the two groups (Table 1). The mean BMI was 22.34 ± 2.60 in the HCs, and 21.02 ± 2.02 in heroin addicts and there was a significant difference (*p* < 0.05) (low nutritional status) between the two groups. The serum levels of D-lactic acid (DLA), endotoxin (ET) and diamine oxidase (DAO) were the main indicators for evaluating the function of intestinal mucosal injury, intestinal wall permeability, and bacterial translocation (5). Compared to HCs, heroin addicts have a higher level of DAO, ET, and DLA (10.97 ± 3.24 U/L vs. 13.54 ± 3.27 *p* < 0.0001; 18.22 ± 3.41 U/L vs. 20.83 ± 5.36 U/L *p* < 0.05; 7.92 ± 4.21 mg/L vs. 10.36 ± 5.01 mg/L *p* < 0.05; respectively, Table 1). These results suggest a potential intestinal injury in the group of heroin addicts of withdrawal stage.

**Table 1:** The clinical information and intestinal barrier indicators of study population.

	**HCs**	**Heroin**	***p*-value**
Subjects	*N* = 38	*N* = 38	NA
Sex	Male	Male	NA
Age (years)	34.44 ± 11.86	34.47 ± 8.40	0.122
BMI (Kg/m^2^)	22.34 ± 2.60	21.02 ± 2.02	0.0162^*^
Diamine oxidase (U/L)	10.97 ± 3.24	13.54 ± 3.27	0.0009^***^
Exotoxin (U/L)	18.22 ± 3.41	20.83 ± 5.36	0.0135^*^
D-lactic acid (mg/L)	7.92 ± 4.21	10.36 ± 5.01	0.0247^*^

*The population demographics and serum levels of diamine oxidase (DAO), exotoxin (ET) and D-lactic acid (DLA) in heroin addicts and healthy controls (HCs). The results represent mean ± SD (p-values were calculated using unpaired t-test)*,

* *p < 0.05;*

*** *p < 0.001*.

### Generation of a Mouse Model With Heroin Dependence

After repeated 10 mg/kg heroin administrations for 14 days, the mouse model with heroin dependence was established and confirmed by behavior sensitization (Figures 1A–D). The total traveled distance collected in the OFT showed that heroin dependent mice traveled a significantly longer distance than control mice (101.09 ± 16.43 vs. 600.95 ± 93.86, *p* < 0.0001 Figures 1B–D), which was considered an addiction behavior (18). Data analysis showed that the body weight of the heroin dependent mice was significantly decreased relative to that of control mice (Figure 1E). Symptoms related to anxiety and/or depression were most often reported in drug withdrawal; herein, after 48 h withdrawal, anxiety and depression-like behaviors were evaluated by OFT, EMP, TST, and FST. The heroin dependent mice spent less time in the center of the open field (*p* < 0.05, Figures 1F,J) and decreased exploration of the open arms of the elevated-plus maze (*p* = 0.07, Figures 1G,K) during the 5-min test compared to mice that had received saline. For the TST and FST, heroin treated mice during withdrawal have reduced mobile time (*p* < 0.0001, Figure 1H) and swim time (*p* < 0.001, Figure 1I). Thus, mice with heroin dependence exhibited worse depressive and anxiety symptoms.

### Disrupted the Intestinal Mucosal Integrity in Mice With Heroin Dependence

In our heroin-dependence mouse model, heroin-induced gut barrier dysfunction may occur at any time within the 21 days during heroin treatment or after the initiation of drug withdrawal. We thus compared the serum level of DLA, ET, and DAO, the expression of Claudin-1 at both day 21(Heroin-addicted stage) and at 48-h after initiation of withdrawal (Heroin-withdrawal stage) (Figure 2A). Compared to non-treated control mice, both heroin-addicted and heroin-withdrawal groups decreased the integrity of the intestinal mucosal barrier, as demonstrated by significant increases in the levels of serum DLA, ET, and DAO (Figures 2B–D), lower expression level of Claudin-1 (Figures 2E,F). These results were consistent with previous analysis in heroin addicts (Table 1). In addition, no significant differences were observed between the heroin-addicted and heroin-withdrawal groups (*p* > 0.05). For better mimicking the heroin dependence and withdrawal syndrome in patients, we decided to profile the microbiota in the heroin dependent mouse model undergoing withdrawal stage to explore the associations with intestinal mucosal integrity.

### Discrepancy in the Gut Microbiota Structure and Diversity Between Heroin-Dependent and Control Groups

**Figure 3:**
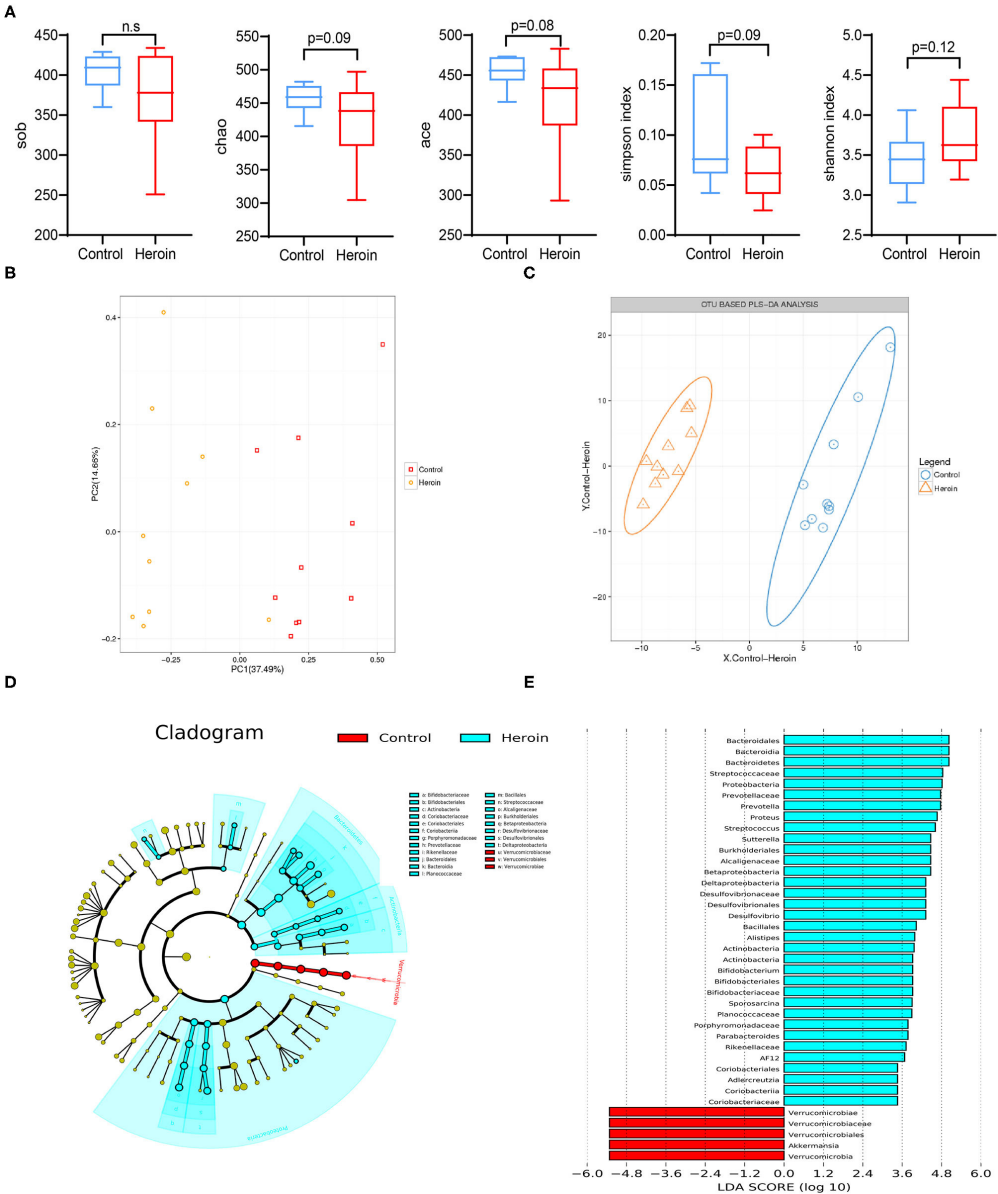
Heroin administrated altered the gut microbiota structure and diversity. **(A)** Comparison of the alpha-diversity indices between the control and heroin groups based on the observed species, chao, ace, shannon, and simpson index. **(B)** Principal coordinate analysis (PCoA) of the samples using Unweighted-UniFrac from pyrosequencing. The red squares represent the control group, and the yellow dots represent the heroin administrated group. The fecal microbiotas of the two groups could be divided into clusters according to community composition using Unweighted UniFrac metrics and separated clearly using PCoA analysis. **(C)** Operational taxonomic unit (OUT)-based partial least-squares discrimination analysis (PLS-DA). **(D)** Linear discriminant analysis effect size (LEfSe) cladogram representing differentially abundant taxa. **(E)** Linear discriminant analysis (LDA) scores, as calculated by the LEfSe of differentially abundant taxa in the two groups. Cladogram showing differentially abundant taxonomic clades with an LDA score >2.0 among cases and controls.

In general, greater bacterial diversity was considered beneficial to health; the gut microbiota in the heroin-dependent group was slightly decreased although no statistical differences were presented based on sob, chao, ace, Shannon, and simpson indices (Figure 3A). The fecal microbiota of the two groups could be divided into clusters according to community composition using Unweighted UniFracmetrics and clearly separated in the PCoA plot demonstrating the structural changes in the microbial communities (Figure 3B). The OUT-based PLS-DA analysis also showed apparent differences between the groups (Figure 3C). The linear discriminant analysis effect size **(**LEfSe) cladogram represents differentially abundant taxa (Figure 3D). *Bacteraidaceae, Prevatellaceae, Ruminococcaceae, Verrucomicrobia*, and *Akkerrnassia* were the key types that contributed to the difference in the microbiota composition between the two groups. The significant difference in bacterial abundance was compared using linear discriminant analysis (LDA). Moreover, the composition of the gut microbiota was significantly different from the two groups according to the LDA score (Figure 3E).

### Heroin Dependence Altered Microbiota Composition in Mice

**Figure 4:**
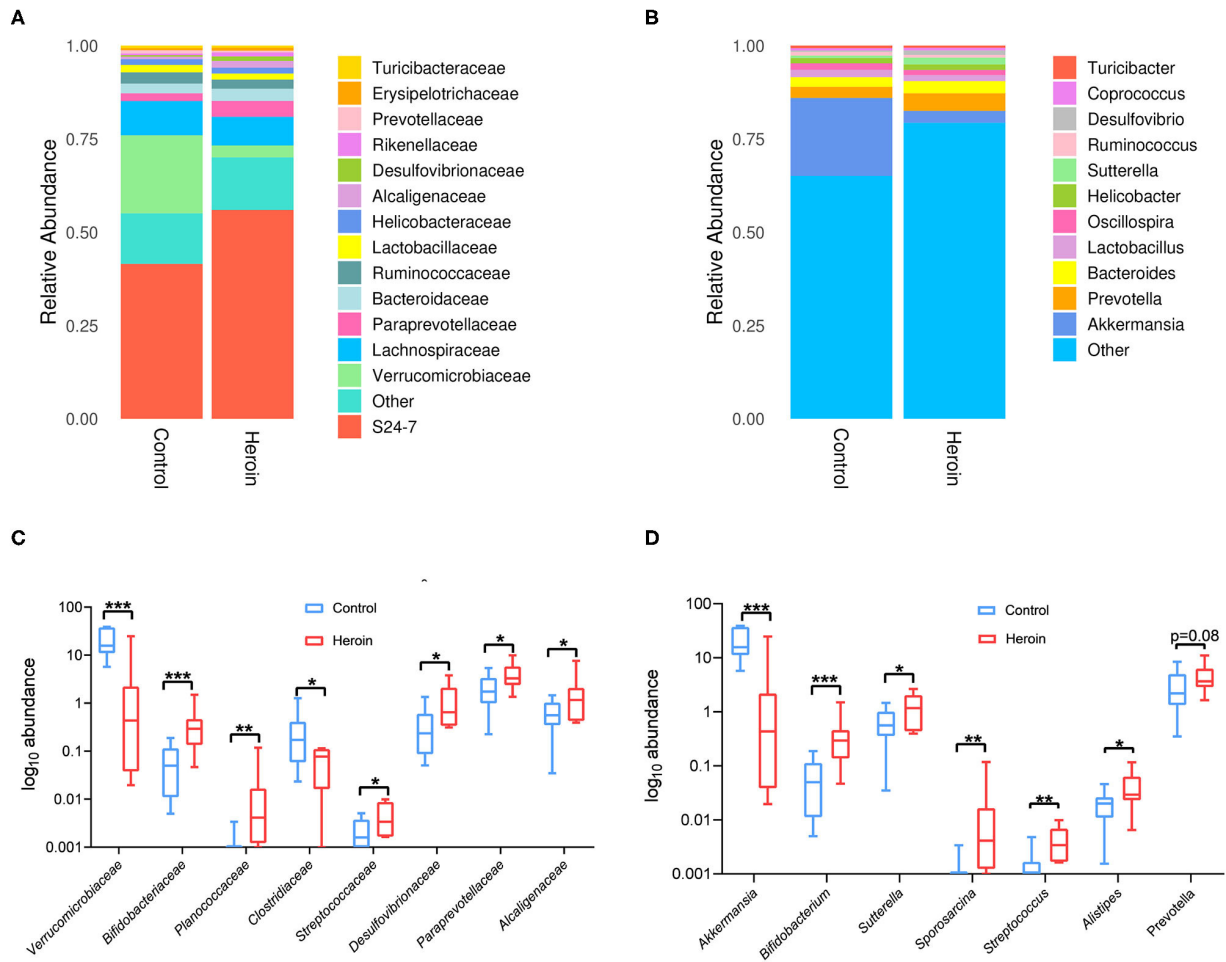
Family and genus-level taxonomic distribution of intestinal microbiota and the top microbiota (the relative abundance >0.01%) at the family and genus levels in the control and heroin groups. **(A,B)** Bacterial community structures in the heroin administrated and control groups at the family **(A)** and genus levels **(B)**. Abundance is presented in terms of the percentage of the total effective bacterial sequences in each group. **(C,D)** All significantly different bacterial are listed in the heroin and control groups at the family **(C)** and genus levels **(D)**. Data are represented as boxplots, with median, minima, and maxima, statistical significance was calculated using Wilcoxon-test (*n* = 10) *p < 0.05; **p < 0.01; ***p < 0.001.

We identified the top microbiota (the relative abundance >0.01%, <0.01% were classified into ‘others’) at the family (Figure 4A) and genera (Figure 4B) levels in the heroin and control groups. At the family-level, the top microbiota includes S24-7, *Verrucomicrobiaceae, Lachnospiraceae, Paraprevotellaceae, Bacteroidaceae, Ruminococcaceae, Lactobacillaceae, Helicobacteraceae, Alcaligenaceae, Desulfovibrionaceae, Rikenellaceae, Prevotellaceae*, and *Erysipelotricchaceae* (Figure 3A). The relative abundances of *Verrucomicrobiaceae, Paraprevotellaceae*, and *Alcaligenaceae* differed between the two groups (*p* < 0.05). Mice with heroin dependence had a higher abundance of *Bifidobacteriaceae, Planococcaceae, Streptococcaceae, Desulfovibrionaceae* (the relative abundance of these bacteria <0.01%) compared to control mice (Figure 4C). At the genus-level, 11 genera were observed in the top microbiota of both groups (the relative abundance >0.01%) (Figure 4B), of these, *Akkermansia* was significantly decreased while *Bifidobacterium* and *Sutterella* were significantly enriched in the heroin group (Figure 4D). The remaining genera were not significantly enriched in either group (*Prevotella, Bacteroides, Lactobacillus, Oscillospira, Coprococcus, Ruminococcaceae, Turicibacter*, and *Helicobacter*). In addition, the level of *Bifidobacterium, Sporosarcina, Streptococcus*, and *Alistipes* (the relative abundance of these bacteria <0.01%) were increased more in heroin dependent group than that in the control group (Figure 4D).

### Altered Gut Microbiota Is Associated With Impaired Intestinal Mucosal Barrier Integrity in Heroin Dependent Mice

**Figure 5:**
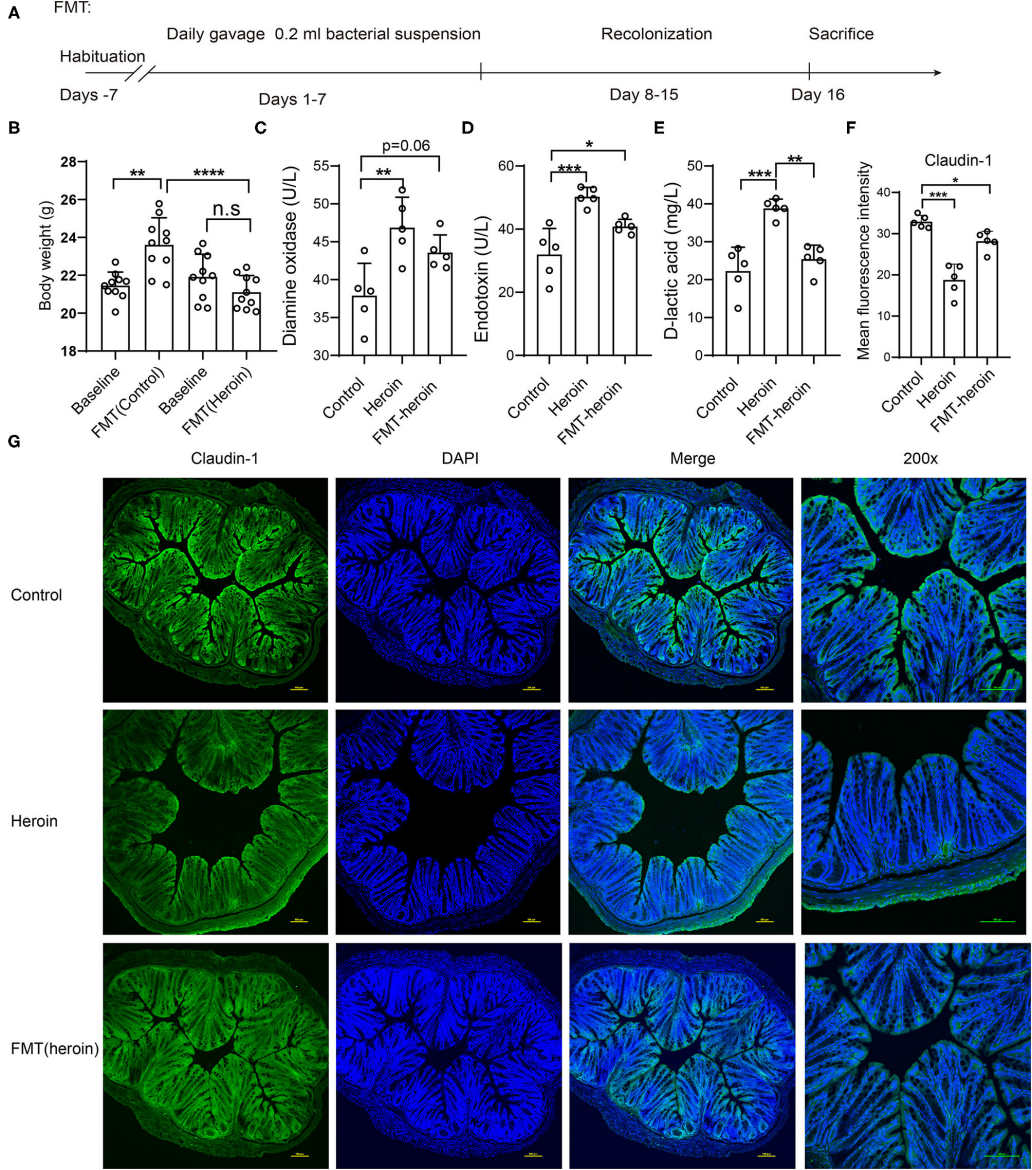
Effect of FMT treatment on intestinal permeability and expression of tight junction proteins in mice. **(A)** Scheme of FMT experiment. **(B)** The body weight of pre-FMT and Post-FMT in receive control microbiota and heroin microbiota groups. **(C–E)** The serum levels of DLA, ET and DAO in control and FMT groups. **(F,G)** Immunofluorescence showed decreased expression levels of Claudin-1 in heroin and FMT groups. **(F)** Mean fluorescence intensity of Claudin-1 expression. **(G)** Example images. All data are expressed as the mean ± SD *p < 0.05; **p < 0.01; ***p < 0.001; ****p < 0.0001.

To investigate whether heroin-dependent altered gut microbiota was sufficient to influence the integrity of the intestinal mucosal barrier in mice, the FMT experiments were performed (Figure 5A). Recipient mice receiving microbiota from heroin dependent mice showed a decreased body weight compared to mice that received FMT from controls (Figure 5B). Significant elevation of serum DAO (*p* = 0.0305) and ET (*p* = 0.0476) were observed in the FMT mice receiving microbiota from heroin dependent mice compared to control mice (Figures 5C,D), although the DLA levels increased was not statistically significant (*p* = 0.36, Figure 5E). The intestinal mucosal barrier integrity was crucial for intestinal homeostasis, and its function was maintained by intercellular TJs. Intestinal TJs, such as OCL and ZO-1 have been shown as the principal determinants of intestinal mucosal barrier integrity (23). Furthermore, we examined the expression and distribution of TJ proteins in the colon using immunofluorescence. As shown in Figures 5F,G, the expression of Claudin-1 and ZO-1 (Supplementary Figure 1) were decreased in both heroin dependence and FMT groups and these results indicated intestinal mucosal mechanical barrier impairment. Taken together, the results showed that heroin dependence induced intestinal mucosal barrier function impairment could be transmissible via gut microbiota in mice.

### Analysis of Fecal Short-Chain Fatty Acids (SCFAs) in Mice

**Figure 6:**
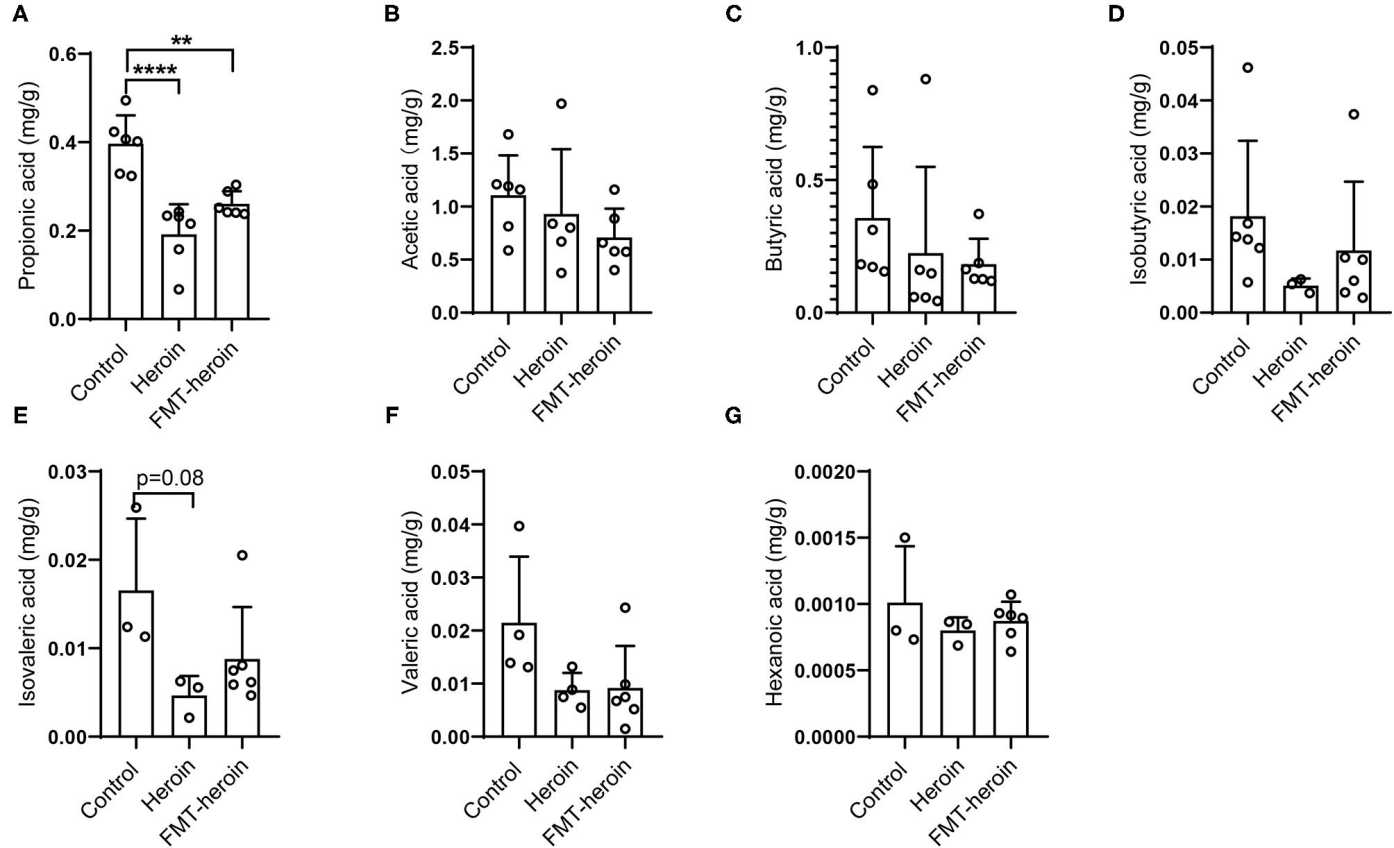
Composition of short chain fatty acids in fecal samples of control, heroin-administrated and FMT treated mice. The levels of propionic acid were significantly altered in the heroin and FMT groups **(A)**. **(B–G)** Concentrations of the major SCFAs acetate, butyrate, isovaleric, isobutyric, valeric, hexanoic. All data are expressed as the mean ± SD (*n* = 6 per group). Statistically differences were analyzed with one-way ANOVA, **p < 0.01; ****p < 0.0001.

SCFAs are the primary products of microbial fermentation of undigested dietary carbohydrates especially acetate, propionate, and butyrate (24). Several reports highlighted the immunomodulatory capacities and the ability to strengthen epithelial barrier integrity (25, 26). The intestinal microbiota and their derived SCFAs are crucial to intestinal barrier integrity. Therefore, the levels of acetic acid, propionic acid, butyric acid, valeric acid, isobutyrate, and isovalerate in the fecal samples were analyzed by GC/MS. Acetic acid, butyric acid and propionic acid were the principal fecal SCFAs present in the mouse model, the concentrations of propionic acid significantly decreased in heroin dependence and FMT mice compared to control mice (Figure 6).

## Discussion

The microbiota-gut-brain axis were considered as the bidirectional system of communication between central nervous system (CNS) and the gastrointestinal tract (27). Many CNS diseases such as autism spectrum disorder, Parkinson's disease, and major depressive disorder are more susceptible to gastrointestinal symptoms disorder (28, 29), even more, gastrointestinal symptoms of individuals seems to strongly correlate with the severity of CNS diseases (28). Gastrointestinal ailments are the most frequent complications in heroin abusers, which not only aggravate the disease process, but even lead to low abstinence rate. The role of microbiota contribute more than 50% to the development of GI symptoms (30). In this study, a significant reduction in body weight was observed in heroin addicts and heroin dependent mice, as well as colonization with heroin dependence-altered microbiota by FMT. As body weight is commonly used to monitor the nutritional status, these results revealed that the gastrointestinal dysfunction-related decline in nutritional status was associated with heroin dependence.

According to the results of 16S rDNA sequencing, there were no significant differences in the alpha diversity, consistent with the results comparing cocaine users and non-users (31). At the genus level, *Akkermansia*, which belongs to the family of *Verrucomicrobia*, was significantly increased in the heroin dependent mouse group (Figures 4C,D). Previous study suggested that higher body weight and lower *Akkermansia* abundance were found in the monosodium glutamate-induced abdominal obesity mice study (32), while in our present study lower *Akkermansia* abundance and lower weight were shown in heroin dependent mice. We speculate that Akkermansia may synergrise with other bacteria and involve in tighten intestinal barrier integrity, lipid storage, adipose tissue inflammation and insulin resistance. Therefore, it is reasonable to believe that the body weight loss was to some extent related to the change of *Verrucomicrobia*. In parallel, it has been shown in previous reports that the abundance of *Akkermansia* may directly exacerbate intestinal inflammation (33). Furthermore, *Akkermansia* was found to be involved in regulating inflammatory factors (34) and played an important anti-inflammatory role in the development of metabolic syndrome (35). Recent human studies have also shown that reduced *Akkermansia* were found in IBD patients (36, 37), and in children with autism spectrum disorder with a thinner GI mucus barrier (38). Mechanistically, *Akkermansia* may lead to dry stool and eventually intestinal mucosal barrier dysfunction by degrading intestinal mucin (32). Therefore, heroin dependence induced *Akkermansia* altering should trigger our more attention in clinical diagnosis and treatment. The relative abundance of *Bifidobacteriaceae* at family and genus levels were increased in mice with heroin dependence (p < 0.001, Figures 4C,D) compared with control mice. However, it is generally agreed that *Bifidobacteriaceae* is considered to be beneficial in humans and animals (39). A recent study showed that the *Bifidobacterium* were significantly elevated in early days while returned to baseline levels after 7 days of methamphetamine cessation in rat model (40). Additionally, a higher level of *Bifidobacteriaceae* was found in major depressive disorder (41). These results align with our findings, and we speculated that increases in relative abundance of these gram-positive bacteria may act as a protective mechanism against heroin acute withdrawal, alternatively, specific strains of *Bifidobacterium* may have inflammatory potential.

*Sutterella* is a genus of the family *Sutterellaceae* which belongs to the class β*-Proteobacteria*, have already proven that low levels are associated with gut immune homeostasis and high levels of IgA (42), meanwhile, low fecal IgA levels were attributed to the presence of high levels of *Sutterella* in a mouse model (43). *Sutterella* could impair the functionality of the intestinal antibacterial immune response, mainly it's capacity to limit intracellular bacterial species, rather than directly induce inflammation (44). Thus, *Sutterella* species are considered as commensals or bystanders in the context of gastrointestinal diseases (45, 46). Herein, we found that mice with heroin dependence exhibited an increased abundance of *Sutterella* which suggest intestinal barrier injury should be focused on. To date, the role of the *Prevotella* genus within the intestinal microbiota and its effects on the host are not entirely understood, whereas somewhat conflicting interpretations are available linking the *Prevotella* genus to health markers. Beneficial effects of some *Prevotella* strains in the gut have been reported, such as cardiovascular disease risk factor profile and glucose metabolism improvement (47, 48). Furthermore, *Prevotella* strain abundance has been found in healthy gut microbiota and was associated with plant-rich diets (49, 50). However, studies have also suggested a potential role of *Prevotella* species as intestinal pathobionts, as some strains promoted diseases, such as metabolic syndrome, obesity, inflammatory bowel disease or other inflammatory diseases (51). In mouse models, *Prevotella* enrichment was positively correlated with high risk of irritable bowel syndrome (IBD) and associated with higher susceptibility to chemically-induced colitis (52) by reducing interleukin (IL)-18 (53). Herein, as one of the top bacteria, *Prevotella* exhibited a relatively higher abundance, although the difference did not reach statistical significance (*p* = 0.08). In addition to the details described above, the altered bacterial such as *Desulfovibrio, Prevotella*, and *Oscillibacter* may utilize microbial exopolysaccharides synthesized by *Bifidobacterium* to produce SCFAs in the intestine (54).

The balance of gut microbiota helps to maintain the biological barrier of the intestine (55). Mice with heroin dependence exhibited several changes in bacterial, which might damage the integrity of the intestinal barrier. To further explore the role of microbiota in the intestinal barrier function, we performed FMT for subsequent studies. Consistent with our expectation, we observed the changes of intestinal mucosal barrier integrity and permeability (Figures 5C–E), further supported by immunofluorescence results of Claudin-1 and ZO-1 (Figure 5F, Supplementary Figure 1). FMT experiments suggest that the alteration of microbiota is closely related to the function change in the intestinal barrier.

In addition, SCFAs are the most critical and pleiotropic functional components of microbe-to-host signaling (56). It is now well-established that SCFAs modulate colonic motility by stimulating serotonin secretion from gut enterochromaffin cells (56, 57) and SCFAs are closely related to intestinal growth and barrier function (58). In the present study, we observed a significant decrease in the concentration of propionic acid in both heroin dependent mice and FMT mice (Figure 6A) compared to controls. Considering propionate can readily cross the gut-blood barrier and has potential health-promoting effects (59–61), we speculate that propionic acid may participate in TJ assembly by regulating TJ proteins and modulating the integrity of intestinal mucosal in mice.

In the current study, the strength of our work is that we utilized FMT mouse model to prove of concept that heroin dependence-altered microbiota is associated with disrupted intestinal mucosal integrity and permeability. However, there are some limitations. First, the results warrant further validation in other independent cohort studies with larger sample sizes. Second, there might be other subtle differences between undergoing heroin exposure and in withdrawal stage. Thus, additional animal models distinguishing between heroin-induced vs. withdrawal-induced damage to intestinal mucosal integrity and gut microbiota will be useful to determine the best timing for preventive intervention for heroin withdrawal management.

In summary, our study provides evidence showing that heroin dependence could alter gut microbiota and modulate mucosal barrier integrity, highlighting the role of the gut microbiota in substance use disorders and the pathophysiology of gastrointestinal disease.

## Data Availability Statement

The datasets presented in this study can be found in online repositories. The 16S rRNA gene sequencing data are available from NCBI Bioproject Accession number: PRJNA770289.

## Ethics Statement

The studies involving human participants were reviewed and approved by the Clinical Research Ethics Committee, the First Affiliated Hospital of Kunming Medical University. The patients/participants provided their written informed consent to participate in this study. The animal study was reviewed and approved by the Animal Care and Use Committee of Kunming Medical University. Written informed consent was obtained from the owners for the participation of their animals in this study.

## Author Contributions

JY and KW: designed the experiments. JY, PX, LB, ZZ, YZ, CC, and ZX: recruited clinical participants and collected samples. YX, MC, MZ, and HW: animal behaviors and mice experiment. JY, KW, and ZZ: analyzed the 16S rRNA and metabolomics data. JY and ZZ: drafted the manuscript. All authors contributed to the article and approved the submitted version.

## Funding

This work was supported by the National Natural Science Foundation of China (Nos. 3171101074, 81870458, and 31860306); Yunling Scholar (YLXL20170002); the Major project of Yunnan Provincial Bureau of Education (2020J0161; 2021J0234); Yunnan Engineering Technology Center of Digestive Disease (2018DH006); and Science and Technology Department of Yunnan Province (Nos. 202001AV070010, 2020DAMARA-004, and 2020DAMARA-005).

## Conflict of Interest

The authors declare that the research was conducted in the absence of any commercial or financial relationships that could be construed as a potential conflict of interest.

## Publisher's Note

All claims expressed in this article are solely those of the authors and do not necessarily represent those of their affiliated organizations, or those of the publisher, the editors and the reviewers. Any product that may be evaluated in this article, or claim that may be made by its manufacturer, is not guaranteed or endorsed by the publisher.

## Abbreviations

BMI: body mass index
DLA: D-lactic acid
DAO: diamine oxidase
EPM: elevated plus-maze test
ET: endotoxin
FMT: fecal microbiota transplantation
FST: forced swimming test
GC-MS: gas chromatography–mass spectrometry
LEfSe: linear discriminant analysis effect size
LDA: linear discriminant analysis
OCL-1: occludin-1
OFT: open-field test
OTUs: operational taxonomic units
PCA: principal component analysis
PLS-DA: partial least-squares discrimination analysis
SCFAs: short chain fatty acids
TJ: tight junction
TST: tail suspension test
ZO-1: zona occludens-1.
