# Supplementary figures and images for: The Association of Altered Gut Microbiota and Intestinal Mucosal Barrier Integrity in Mice With Heroin Dependence
Source: Front Nutr. 2021 Nov 4;8:765414. doi: 10.3389/fnut.2021.765414 (PMC8600332; doi:10.3389/fnut.2021.765414)

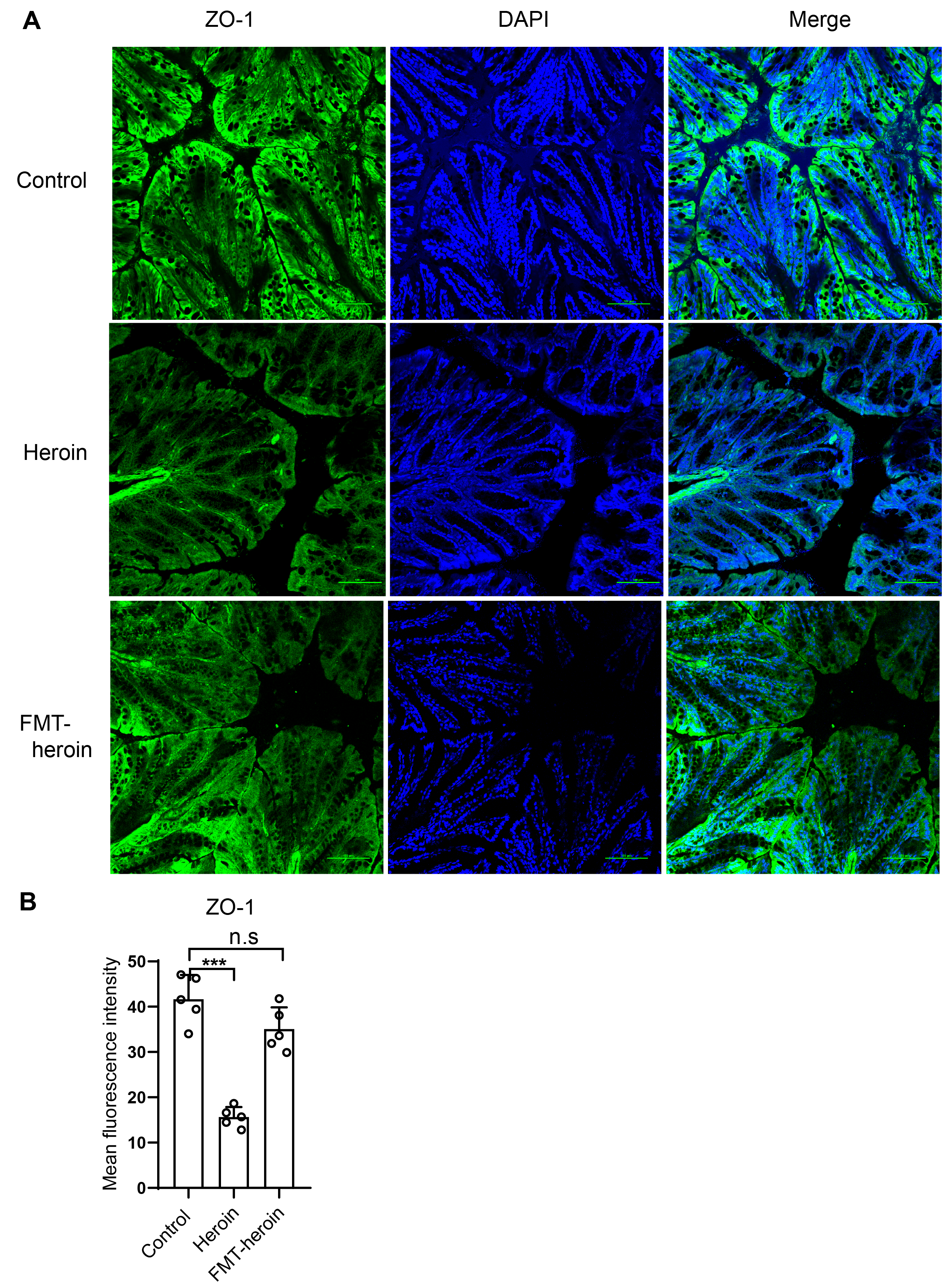

Supplement: Supplementary file 1 [file Image_1.tif]
